# Simultaneous p53 and p16 Immunostaining for Molecular Subclassification of Head and Neck Squamous Cell Carcinomas

**DOI:** 10.1007/s12105-024-01680-z

**Published:** 2024-08-07

**Authors:** Pihla Pakkanen, Antti Silvoniemi, Katri Aro, Leif Bäck, Heikki Irjala, Leena-Maija Aaltonen, Jaana Hagström, Caj Haglund, Jukka Laine, Heikki Minn, Jutta Huvila

**Affiliations:** 1 Department of Otorhinolaryngology - Head and Neck Surgery, University of Helsinki, Helsinki University Hospital, Helsinki, Finland; 2 Department of Otorhinolaryngology - Head and Neck Surgery, University of Turku and Turku University Hospital, Turku, Finland; 3 Department of Pathology, University of Helsinki and Helsinki University Hospital, Helsinki, Finland; 4 Department of Oral Pathology and Radiology, University of Turku, Turku, Finland; 5 Translational cancer research program unit, Faculty of Medicine, University of Helsinki, Helsinki, Finland; 6 Department of Surgery, Helsinki University Hospital and Helsinki University, Helsinki, Finland; 7 Department of Pathology, University of Turku, Turku University Hospital, D5020, Medisiina D, 5. floor Kiinamyllynkatu 10, Turku, FIN-20520 Finland; 8 Department of Oncology, University of Turku, Turku University Hospital, Turku, Finland

**Keywords:** Head and neck SCC, HPV-associated, HPV-independent, p16, p53

## Abstract

**Purpose:**

Our aim was to assess the ability of simultaneous immunohistochemical staining (IHC) for p16 and p53 to accurately subclassify head and neck squamous cell carcinomas (HNSCC) as HPV-associated (HPV-A) versus HPV-independent (HPV-I) and compare p53 IHC staining patterns to *TP53* mutation status, p16 IHC positivity and HPV status.

**Methods:**

We stained 31 HNSCCs for p53 and p16, and performed next-generation sequencing (FoundationOne©CDx) on all cases and HPV in-situ hybridization (ISH) when sufficient tissue was available (*n* = 23). p53 IHC staining patterns were assessed as wildtype (wt) or abnormal (abn) patterns i.e. overexpression, null or cytoplasmic staining.

**Results:**

In a majority of cases (28/31) interpretation of p16 and p53 IHC was straightforward; 10 were considered HPV-A (p16+/p53wt) and 18 cases were HPV-I (p16-/p53abn). In the remaining three tumours the unusual immunophenotype was resolved by molecular testing, specifically (i) subclonal p16 staining and wild type p53 staining in a tumour positive for HPV and with no *TP53* mutation (HPV-A), (ii) negative p16 and wild type p53 staining with a *TP53* mutation and negative for HPV (HPV-I), and (iii) equivocally increased p16 staining with mutant pattern p53 expression, negative HPV ISH and with a *TP53* mutation (HPV-I).

**Conclusion:**

Performing p16 and p53 IHC staining simultaneously allows classification of most HNSCC as HPV-A (p16 +, p53 wild type (especially basal sparing or null-like HPV associated staining patterns, which were completely specific for HPV-A SCC) or HPV-I (p16 -, p53 mutant pattern expression), with the potential for limiting additional molecular HPV or mutational testing to selected cases only.

## Introduction

After the recognition of the role of human papilloma virus (HPV) in the pathogenesis of cervical squamous cell carcinoma, the association with HPV has been described in many carcinomas; currently it is estimated that 5% of carcinomas are caused by HPV [1]. In the current 5th edition of WHO Blue Book series on classification of human tumours, six carcinoma types are subclassified as HPV-associated (HPV-A) or HPV-independent (HPV-I), based on their HPV status: cervical, vaginal and vulvar squamous cell carcinomas, cervical adenocarcinomas, penile squamous cell carcinomas and oropharyngeal squamous cell carcinoma [2–4].

Head and neck carcinomas affect up to 890 000 people annually [5] and the majority are squamous cell carcinomas (SCC). SCCs in the oropharynx (OPSCC) are subclassified as HPV-A or HPV-I [2]. Additionally, an association with HPV has also been described in nasopharyngeal carcinomas [6], laryngeal carcinomas [7] as well as in sinonasal carcinomas [8]. A widely used method to determine HPV status is performing p16 immunohistochemical staining (IHC) on tumour tissue. p16^ink4a^ is a well-known surrogate marker that is overexpressed in HPV-driven disease and has shown good correlation with HPV status determined by other methods [9–11]. There is accumulating evidence that HPV-A OPSCCs are associated with a significantly better prognosis than HPV-I tumours and the clinical management is different, hence HPV association is routinely determined in OPSCCs and even TNM classification is different for HPV-A and HPV-I tumours [12].

The classification of HPV-A / HPV-I SCC is not limited to oropharyngeal SCCs but applies to all sites where HPV-A SCCs occur. There has also been increasing interest in further subclassification of vulvar HPV-I SCCs according to p53 status as HPV-I (p53abn) if there is mutant pattern p53 expression, and HPV-I (p53wt) SCC if there is wild type p53 expression [13, 14], as the HPV-I (p53wt) SCC have a prognosis that is intermediate between the favourable prognosis of HPV-A SCC and the more aggressive HPV-I (p53abn) SCC [15, 16]. The proportion of SCC in these 3 categories (i.e. HPV-A, HPV-I (p53wt) and HPV-I (p53abn)) vary between different body sites and there is very significant geographical variation [13]. Further work is needed to determine if these 3 categories have differences in their clinical course and disease outcome for body sites other than vulva.

In order to classify HNSCCs into these three different categories, it is crucial that the interpretation of the p16 and p53 IHC staining patterns is done according to current guidelines. According to the College of American Pathologists, p16 is considered positive if over 70% of tumour cells are positive [17]. The pioneering work in the interpretation of p53 staining in SCC has been done in vulvar SCCs and these staining patterns have later been shown to be applicable also in penile, anal and oropharyngeal SCC as well as oral squamous dysplasia [15, 18–22].

Our aim was to study the immunohistochemical (IHC) staining patterns of p53 in head and neck squamous cell carcinomas (HNSCC), and to compare p53 IHC staining patterns to *TP53* mutation status as well as to p16 IHC staining and assess the ability of this two marker IHC panel to consistently and accurately subclassify HNSCC.

## Materials and Methods

### Study Patients

The study was conducted at Turku University Hospital and Helsinki University Hospital, Finland, between 2019 and 2022. A prospective patient cohort participating in an observational ctDNA evaluation study was available for this study. The detailed inclusion and exclusion criteria of the study patients are reported previously [23]. In brief, newly diagnosed patients with stage III-IV HNSCC other than cutaneous, sinonasal and salivary gland carcinoma were eligible for the study. Patients with oropharyngeal p16-positive T3 SCC (stage II) were also eligible. Written informed consent was a prerequisite for participation. The study was conducted in accordance with the latest version of the Helsinki Declaration. The study protocol was approved by the Ethics Committee of the Hospital District of Southwest Finland. Turku and Helsinki University Hospitals granted institutional research permissions.

### Immunohistochemistry

**Fig. 1:**
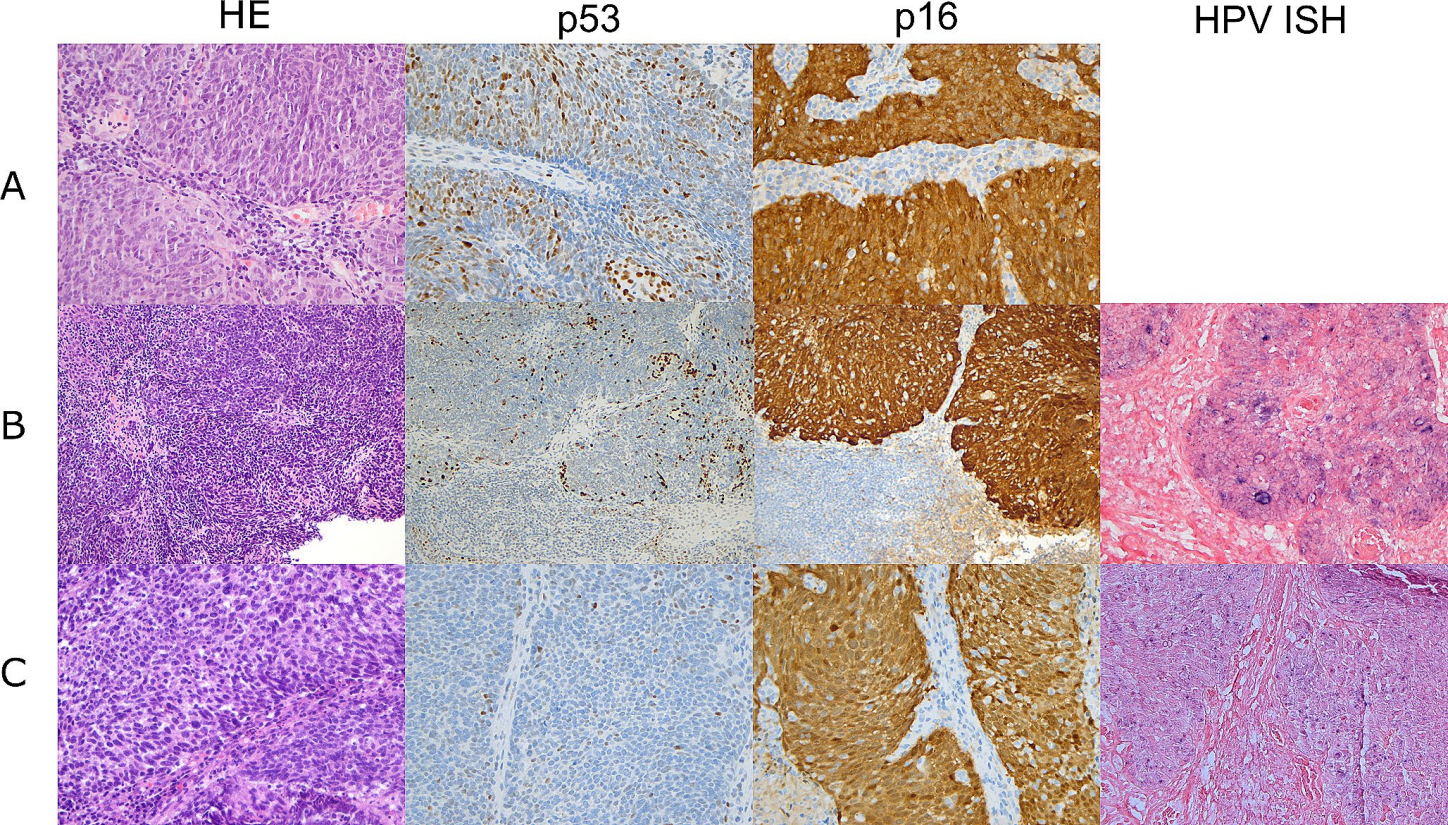
Typical p53 staining patterns associated with HPV-associated p16 positive squamous cell carcinomas, with basal sparing p53 staining pattern (row **A**), scattered positivity with some small clusters (row **B**) and null-like pattern with single positive cells (row **C**). All cases are p16 and HPV ISH positive (when available)

**Fig. 2:**
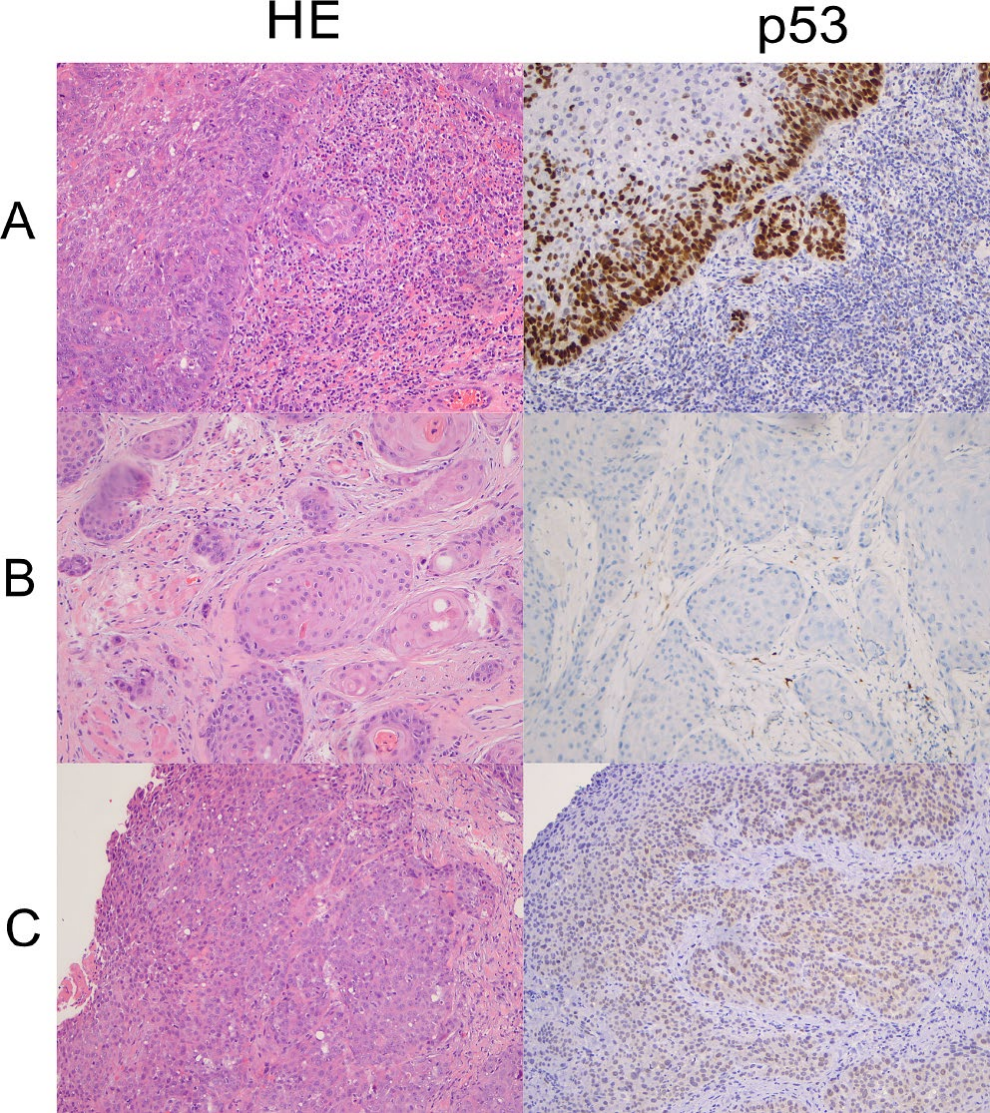
Mutant pattern p53 immunohistochemical staining associated with a *TP53* mutation on NGS. Parabasal p53 expression (row **A**), null-type complete absence of p53 expression (row **B**), and weak cytoplasmic p53 expression (row **C**). All cases were p16 negative

**Fig. 3:**
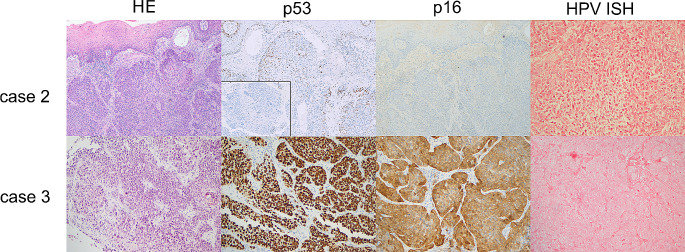
Figure depicts two cases with unusual p16/p53 immunostaining results. Tumour tissue in case 2 shows mostly wildtype p53 staining pattern, with focal null-type staining; the tumour is p16 and HPV ISH negative, and two different *TP53* mutations were detected on NGS. Tumour tissue in case 3 had diffuse p53 overexpression, variable, mostly cytoplasmic 16 positivity and was HPV ISH negative, with a *TP53* mutation detected on NGS. Both cases were categorized as HPV-independent SCC

**Fig. 4:**
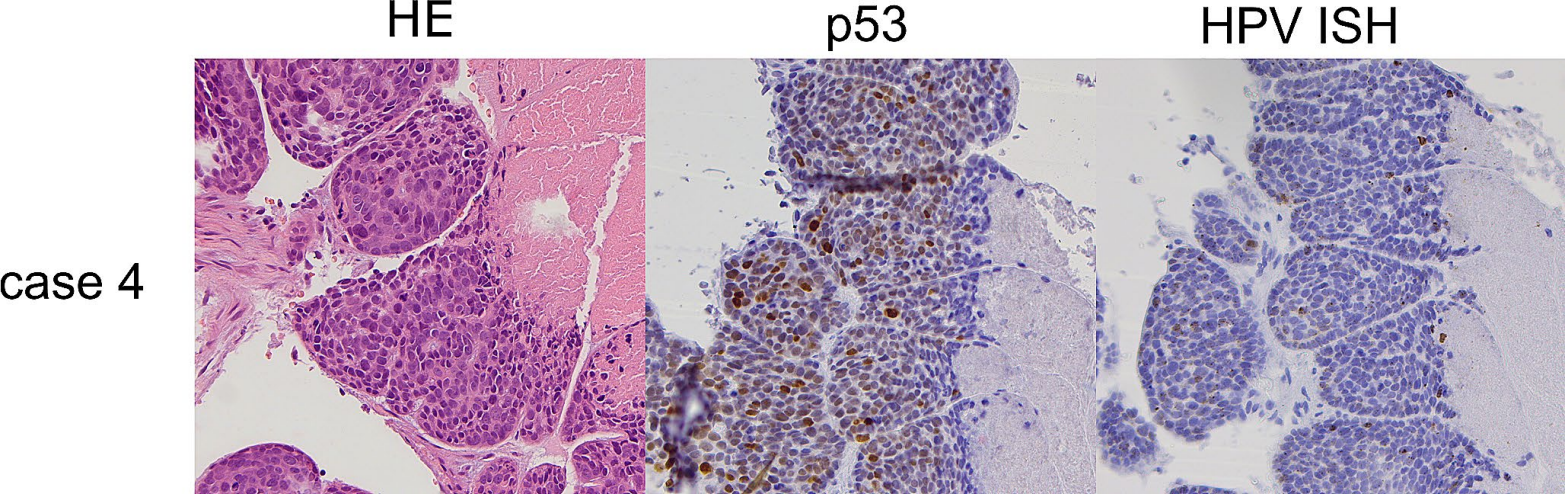
Figure depicts case 4 that showed basal sparing pattern of HPV-associated wildtype p53 staining and was HPV RNA ISH positive. The tumour was also p16 positive and harboured a *TP53* mutation on NGS and was considered to be HPV-A SCC

p16 immunohistochemistry (IHC) was done as part of routine clinical care in all cases and p53 IHC was subsequently performed for this study. Slides of paraffin-embedded tumour samples were cut at 4 microns thickness and immunohistochemical labelling was performed on the Ventana Benchmark Ultra platform automated stainer (Ventana Medical Systems, Tucson, AZ), p16 with a pre-diluted antibody clone E6H4 and p53 with clone Bp53-11 (Ventana, Oro Valley, AZ). p16 was considered positive when 70% or more of tumour cells showed diffuse and strong nuclear and cytoplasmic staining (Fig. 1) and p53 immunohistochemistry was interpreted according to current practice in HPV-I and HPV-A squamous cell carcinoma [21, 24, 25], as showing wildtype or mutant expression pattern, with the latter characterized by diffuse or parabasal overexpression, basal overexpression, null, or cytoplasmic patterns (Figs. 2, 3 and 4). A description of wildtype type staining pattern was recorded as either “conventional wildtype” or “HPV-associated wildtype”, i.e. basal sparing staining or null-like with single positive cells or positive cell clusters, as described previously [18]. p16 interpretation was re-evaluated by three pathologists (JL, JHA, JHU). The interpretation of p53 staining patterns was scored by all three pathologists (JL, JHA, JHU) blinded to the clinical data and p16 status, whereafter they were reviewed with data on p16 status. Discrepant cases were re-evaluated together, and a consensus was reached for all cases. p53 mutational status assessment required a consensus review in 6/31 cases (19.4%).

### In Situ Hybridization

In the Turku cases (*n* = 21) high-risk HPV DNA ISH was performed in a subset of cases as part of clinical care (*n* = 17) and in all remaining p16 positive tumours (*n* = 4) where there was sufficient tissue; this was done using the Roche INFORM HPV III Family 16 probe set (Ventana Medical Systems, Tucson, AZ, USA) that captures HPV genotypes 16, 18, 31, 33, 35, 39, 45, 51, 52, 56, 58 and 66. Signals were detected with the ISH iView Blue Plus Detection Kit (Ventana Medical Systems, Tucson, AZ, USA). All reagents were provided pre-diluted and ready-to-use on BenchMark Series automated slide stainers (Ventana Medical Systems, Tucson, AZ). Punctate hybridization signals localized to the tumour cell nuclei defined an HPV-positive tumour. An HPV positive oropharyngeal cancer was used as a positive control.

### mRNA in Situ Hybridization

For the Helsinki cases (*n* = 10) high risk HPV was detected using E6/E7 mRNA ISH, performed using the RNAscope^®^ 2.5 HD Reagent kit (Advanced Cell Diagnostics, Inc, Hayward, CA) according to the manufacturer’s protocol. HR HPV 18 cocktail probe (RNAscope^®^ Advanced Cell Diagnostics, Inc, Hayward, CA) for genotypes 16, 18, 26, 31, 33, 35, 39, 45, 51, 52, 53, 56, 58, 59, 66, 68, 73, and 82 was used for hybridization. An endogenous housekeeping gene HS-PPIB (RNAscope^®^ Advanced Cell Diagnostics, Inc, Hayward, CA) probe was used as a positive control and a bacterial gene DapB, diaminopimelate (RNAscope^®^ Advanced Cell Diagnostics, Inc, Hayward, CA) probe as a negative control. The staining was scored as follows; a finding of one or more punctate brown nuclear or cytoplasmic dots per tumour cell was regarded as a positive staining result (Fig. 1). The scoring was performed independently by two researchers (JHA and JHU) and consensus was reached.

### Genomic Studies

Tissue genomic analysis was performed using an NGS-based FoundationOne^®^ CDx test assay detecting 324 genes, including 309 genes with complete exonic coverage [26]. The test also reported a variant allele frequency for each detected variant. The histopathologic tumour sample for tDNA analysis was obtained when the diagnostic biopsy was performed, before the start of oncologic treatment. Representative paraffin-embedded tumour tissue for genomic profiling was chosen by pathologists (JLA, JHA) and ten 5 μm thin sections were sent for analysis to Foundation Medicine^®^ Laboratories (Cambridge, MA, USA, or Penzberg, Germany).

## Results

**Fig. 5:**
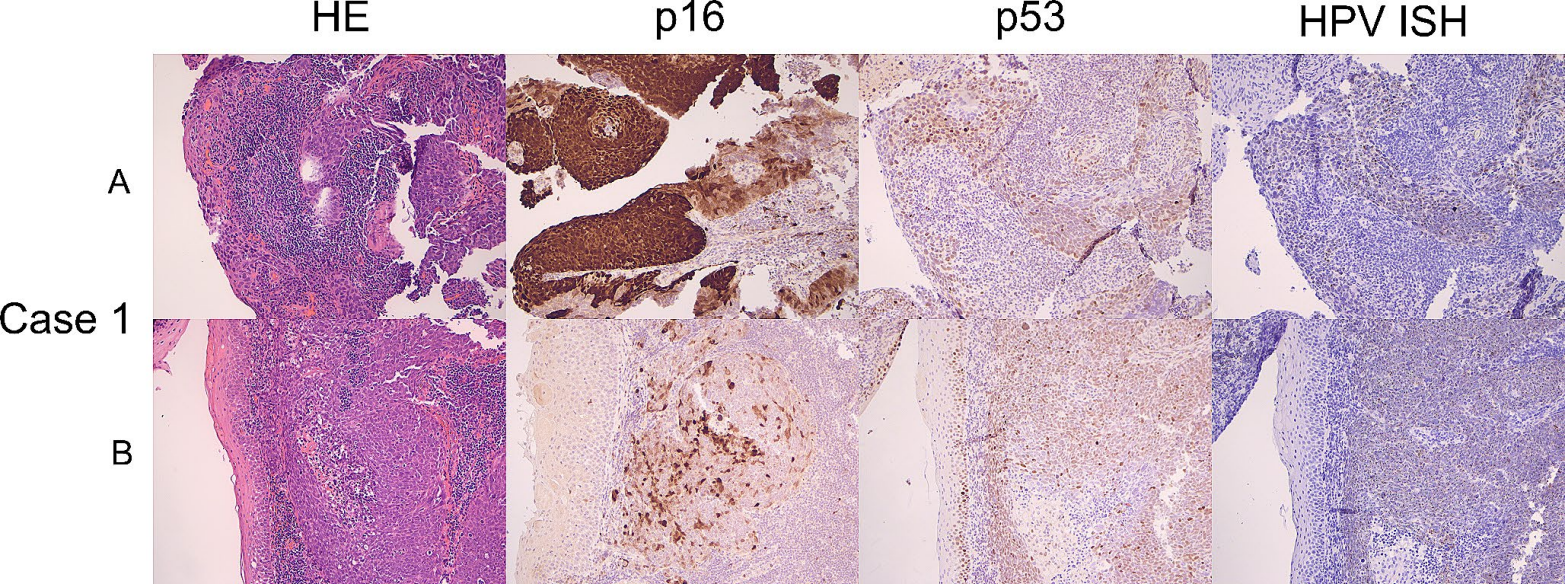
Figure depicts two different tumour foci from case 1. Upper row (**A**) depicts the p16 positive and HPV ISH positive carcinoma on the surface. p53 is wildtype. Lower row (**B**) depicts the invasive component within the same biopsy that shows patchy p16 staining and HPV ISH positivity

Of the 31 tumours in this study, 11 (35.5%) were p16 positive. Of these 11 cases HPV DNA or RNA ISH was available in nine and all were positive (Fig. 1). In one additional case (Case 1) p16 was positive in the surface of the lesion but the invasive component was p16 negative and both components were strongly HPV RNA ISH positive (Fig. 5) and hence it was considered to be HPV-A SCC. Of these 12 HPV-A SCCs all showed wildtype p53 staining and in 10/12 the p53 wild type staining showed features described in HPV-A disease, i.e. basal sparing pattern, small clusters or single positive cells (null-like pattern) (Fig. 1). In one case (Case 4, Fig. 4) a *TP53* mutation was identified on NGS, but the IHC pattern was consistent with HPV-associated p53 staining. Additionally, p16 and RNA ISH were positive, and this case was considered to be HPV-A with a secondary *TP53* mutation.

Of the remaining cases, 19 were p16 negative, and where HPV DNA or RNA ISH was available (*n* = 13), all were HPV ISH negative. Eighteen of 19 showed abnormal mutant expression pattern p53 staining on IHC; 12 overexpression and six null pattern expression of p53. These 18 HPV-I cases harboured one (*n* = 14) or two (*n* = 4) *TP53* mutations. The remaining case (Case 2) was both p16 and HPV ISH negative and the tumour showed p53 wildtype IHC expression but two *TP53* mutations were detected on NGS. p53 IHC was repeated on another tissue block and an abnormal null-type staining pattern in a subclonal distribution was identified (Fig. 3). One of the p16 negative/HPV-I tumours showed increased p16 staining but short of what would qualify as p16 positivity by the criteria used (Fig. 3) and this tumour, which showed abnormal mutant pattern p53 expression, was negative for HPV.

**Fig. 6:**
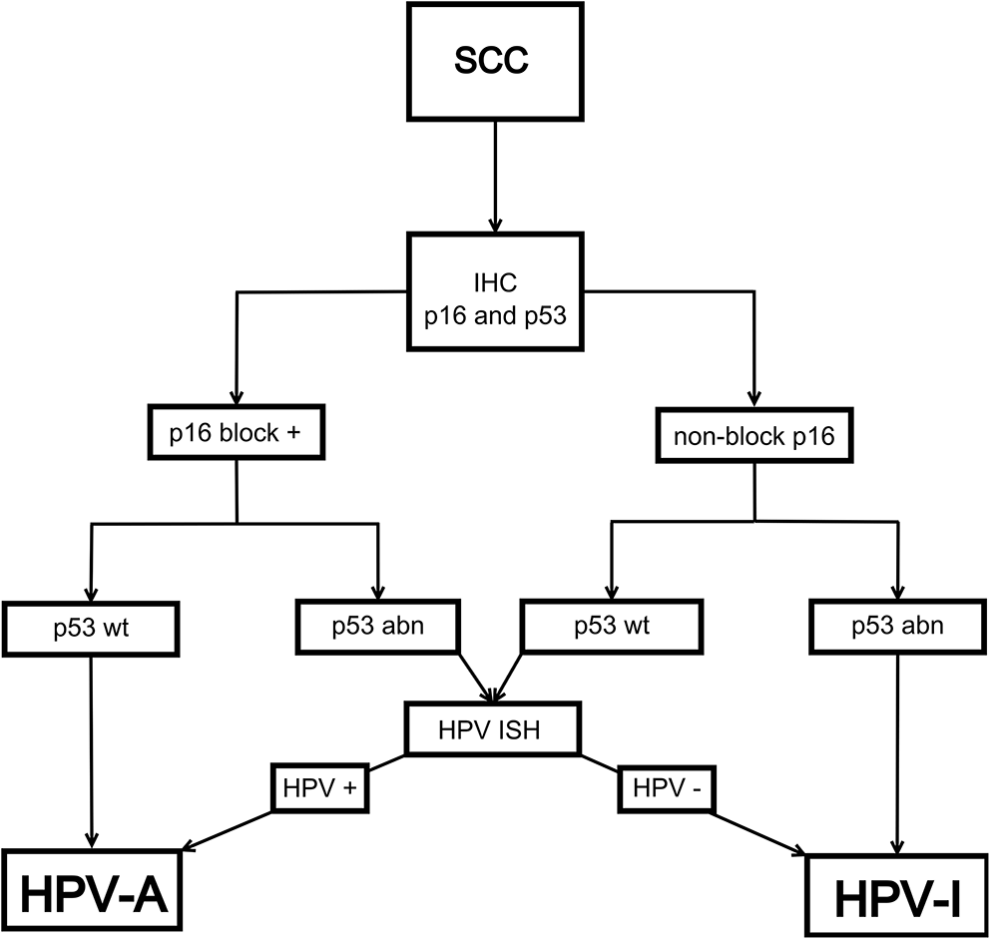
The algorithm used to classify head and neck SCCs into HPV-A and HPV-I carcinomas

In 28 cases the combination of p16 and p53 immunostaining provided an unequivocal classification as HPV-A (p16+/p53 wild type) or HPV-I (p16-/p53 mutant pattern), while in only 3 cases were the p16 and p53 staining results anything other than unequivocal p16 +, p53 wild type or p16 -, p53 mutant pattern expression. Using a proposed algorithm to classify HNSCCs into HPV-A and HPV-I (Fig. 6, based on our results and previous work of others [14]) all cases could be classified as HPV-A or HPV-I (Table 1). The majority, 61.3% of the HNSCCs in our series were HPV-I including all tumours originating in the oral cavity, larynx and hypopharynx. Eleven out of twelve HPV-A tumours arose in the oropharynx where 54.5% of the tumours were HPV-A and 45.5% HPV-I. Adding p53 IHC to p16 staining increased the positive predictive value (PPV) of p16 positivity being indicative of HPV-A disease from 91.7 to 100%. Although the interpretation of p53 can be challenging, ten out of 12 HPV-A showed HPV-associated wildtype p53 staining and the p53 staining patterns associated with high-risk HPV infection (basal sparing or null-like) were strongly associated with HPV-A SCC (sensitivity 83%, specificity 100%).

**Table 1:** Characteristics of HPV-associated and HPV-independent head and neck squamous cell carcinomas

Topography (case#)	p16	HPV ISH	p53 IHC	p53 pattern	TP53 status	TP53 mutation(s)	Final classification
Oropharynx (1)	Subclonal	Pos	Wildtype	Wildtype	no *TP53* mut		HPV-associated
Oropharynx	p16 pos	Pos	Wildtype	Wildtype	NA		HPV-associated
Oropharynx	p16 pos	Pos	Wildtype	Null-like	NA		HPV-associated
Oropharynx	p16 pos	Pos	Wildtype	Basal sparing	no *TP53* mut		HPV-associated
Oropharynx	p16 pos	Pos	Wildtype	Basal sparing/clusters	no *TP53* mut		HPV-associated
Oropharynx	p16 pos	Pos	Wildtype	Basal sparing	no *TP53* mut		HPV-associated
Oropharynx (4)	p16 pos	Pos	Wildtype	Basal sparing	*TP53* mut	D281E	HPV-associated
Oropharynx	p16 pos	NA	Wildtype	Null-like	no *TP53* mut		HPV-associated
Oropharynx	p16 pos	NA	Wildtype	Basal sparing	no *TP53* mut		HPV-associated
Oropharynx	p16 pos	Pos	Wildtype	Clusters	no *TP53* mut		HPV-associated
Oropharynx	p16 pos	Pos	Wildtype	Basal sparing	no *TP53* mut		HPV-associated
Oral cavity	p16 pos	Pos	Wildtype	Clusters	no *TP53* mut		HPV-associated
Oropharynx	p16 neg	Neg	Abnormal	Weak cytoplasmic	*TP53* mut	P316fs*16	HPV-independent
Oropharynx	p16 neg	Neg	Abnormal	Null	*TP53* mut	L257fs*90	HPV-independent
Oropharynx	p16 neg	Neg	Abnormal	Null	*TP53* mut	K139fs*25	HPV-independent
Oropharynx	p16 neg	Neg	Abnormal	Null	*TP53* mut	L257fs*88,	HPV-independent
Oropharynx	p16 neg	Neg	Abnormal	Null	*TP53* mut	Q136*	HPV-independent
Oropharynx (2)	p16 neg	Neg	Abnormal	Subclonal null	*TP53* mut	K120R, T155fs*19	HPV-independent
Oropharynx	p16 neg	Neg	Abnormal	Overexpression	*TP53* mut	S127Y, E258K	HPV-independent
Oropharynx	p16 neg	Neg	Abnormal	Overexpression	*TP53* mut	C242R	HPV-independent
Oropharynx	p16 neg	NA	Abnormal	Overexpression	*TP53* mut	R248Q	HPV-independent
Oropharynx	p16 neg	NA	Abnormal	Overexpression	*TP53* mut	R249S	HPV-independent
Oral cavity	p16 neg	Neg	Abnormal	Overexpression	*TP53* mut	H193N	HPV-independent
Oral cavity	p16 neg	Neg	Abnormal	Overexpression	*TP53* mut	G279E	HPV-independent
Oral cavity	p16 neg	Neg	Abnormal	Overexpression	*TP53* mut	R273C	HPV-independent
Larynx, hypopharynx	p16 neg	NA	Abnormal	Null	*TP53* mut	F212fs*3, splice site 673-1G > A	HPV-independent
Larynx, hypopharynx (3)	Equivocal	Neg	Abnormal	Overexpression	*TP53* mut	Y205C	HPV-independent
Larynx, hypopharynx	p16 neg	NA	Abnormal	Overexpression	*TP53* mut	S241F	HPV-independent
Larynx, hypopharynx	p16 neg	Neg	Abnormal	Overexpression	*TP53* mut	E285K, C242S	HPV-independent
Larynx, hypopharynx	p16 neg	NA	Abnormal	Overexpression	*TP53* mut	R273H	HPV-independent
Larynx, hypopharynx	p16 neg	NA	Abnormal	Overexpression	*TP53* mut	Y205C	HPV-independent

mut = mutation, HPV = human papilloma virus, ISH = in situ hybridization, neg = negative, pos = positive, NA = not assessed

## Discussion

The classification of SCC into HPV-A and HPV-I has become an integral part of the initial diagnostic work up of oropharyngeal SCC, as well as SCC at other sites (gynaecological, penile, and anal) where it has a significant impact on patient management, and thus routine testing for HPV status is warranted. p16 has been shown to be a good surrogate marker for high risk HPV-associated SCC [9–11]. Patients with oropharyngeal cancer and discordant p16 and HPV molecular testing results (p16-/HPV+ (3.8%) or p16+/HPV- (5.6%)) have a significantly worse prognosis than patients with p16+/HPV + oropharyngeal cancer but better than patients with p16-/HPV- oropharyngeal cancer [27]. This highlights the limitations of either p16 IHC or HPV nucleic acid-based testing as a single test to establish HPV status in OPSCC, as neither is completely sensitive and specific [27]. Although guidelines have accepted just p16 testing as sufficient to determine HPV status, consideration is being given to revising the guidelines such that p16 alone is not considered sufficient but should be performed together with high risk HPV testing, which is expensive especially when HPV RNA ISH is performed.

Currently, p53 status does not have an established role in the diagnostic work up of HNSCC, although *TP53* mutations have been identified in over 50% of head and neck carcinomas and their presence was associated with adverse outcomes [28]. In their work Karpathiuo et al. [29] compare the expression of p16 and p53 (null, ≥ 50% positive) IHC in HNSCC and show that 13 out of 25 (52%) p16 positive SCCs are p53 abnormal. While a subset of these may represent “double positives” (discussed later) a majority of these are likely to be misinterpretation of HPV-associated p53 staining patterns such as null-like or basal sparing. The interpretation of p53 IHC has been highly variable in the past [21, 29–31] to the extent that it has prevented meta-analysis [30]; results of earlier studies on p53 IHC cannot be considered reflective of underlying *TP53* mutations. Our results show that there is excellent correlation between mutant pattern abnormal p53 IHC staining and the presence of a *TP53* mutation if the current p53 interpretation guidelines, developed and validated for use in vulvar SCC, are used [24, 25]. The p53 IHC interpretation guidelines specific for HPV-I SCCs have now been reported in vulvar, oropharyngeal and penile SCCs [19, 21, 24, 25] and should be adopted for research and clinical purposes to ensure the quality of IHC data and reliable assessment of the prognostic significance of p53 in HPV-I SCCs in future studies. There is the potential for p53 and p16 staining to be complimentary, providing redundancy and improving accuracy when performed together to determine HPV status; this could overcome some or all of the challenges of using p16 as a single immunomarker, where both false positive and false negative results have been reported in HNSCC.

In our series 28/31 cases were readily classifiable as HPV-A or HPV-I, based on concordant p16 and p53 staining results i.e. either strong diffuse p16 immunoreactivity with p53 showing a wild type staining pattern or p16 negativity with mutant pattern p53 staining. In the remaining three cases additional testing was needed for accurate classification. In one case (Case 3) there was equivocal p16 staining in association with abnormal (mutant-pattern) p53 staining and this case was classified as HPV-I based on negative HPV ISH and the presence of a *TP53* mutation. Although p16 positivity and p53 abnormal pattern are almost mutually exclusive, mutations in *TP53* can occasionally be associated with p16 positivity and lead to misclassification as HPV-A HNSCC. This phenomenon of p16 and p53 “double positivity” is common in some tumour types, such as tubo-ovarian high-grade serous carcinoma but has in addition been described at body sites where HPV-A SCCs occur [15, 18, 20, 21, 32]. In vulvar SCC most of these “double positives” harbour *TP53* mutations and lack evidence of high risk HPV and are thus considered as HPV-I (p53abn) SCCs. The p16 positivity is considered as a secondary overexpression related to p53 loss of function, in a mechanism identical to that seen in high-grade serous carcinoma of tubo-ovarian origin. In oropharyngeal SCC, 8/9 cases with “double positive” p16/p53 staining were negative for HPV [21]. In oropharyngeal SCCs, 2–20% of p16 positive cases are high risk HPV negative [21, 33] and in the recent meta-analysis, p16+/HPV- tumours are shown to have worse 5-year overall survival and 5-year disease-free survival than p16+/HPV+ tumours (81.1% vs. 54.7%; 84.3% vs. 67.9%) [27]. It is likely that many or most of these are explained by presence of a *TP53* mutation in an HPV-I tumour, and these tumours can be detected by staining for both p53 and p16, and accurately classified by HPV RNA ISH. Treating these p16+/HPV- tumours as HPV-A may lead to less intensive treatment protocols and thus worse outcomes.

In our series one case (Case 1) was p16-/HPV+/p53wt and only the superficial component of the tumour was p16 positive, with expression lost in the more deeply invasive component. This case had weak wt p53 staining and did not have a *TP53* mutation, a *CDKN2A* mutation or other mutations that could explain the loss of p16 expression, and it was hypothesized to most likely be due to epigenetic silencing. A similar phenomenon has been previously described [34] and is a potential pitfall that could potentially lead to misclassification of HPV-A disease as HPV-I. It is recommended that where possible, a tumour tissue sample containing superficial tumour rather than deeply invasive tumour or tumour from a metastatic site should be used for p16 IHC, to increase the likelihood of detecting p16 overexpression, as it may be lost during tumour progression. In this case the combination of wildtype p53 and focally positive p16 IHC provided sufficient information leading to additional testing and correct classification as HPV-A.

One challenging case (Case 2) was a p16-/p53wt case, which was HPV ISH negative and showed two different *TP53* mutations on NGS. It is known that p53 IHC is only approximately 95% sensitive for TP53 mutation in tubo-ovarian high-grade serous carcinoma [35]; in these tumours the p53 protein is present at levels comparable to what is seen in tumours without a *TP53* mutation, resulting in wild type staining pattern but an inactive protein. In the case we report, the negative p16 staining provides the clue that the p53 staining is a false negative result and there is an underlying *TP53* mutation, and leads to confirmatory sequencing of *TP53*, another example of the complementary nature of p53 and p16 IHC. In the final challenging case (Case 4) a *TP53* mutation was identified on NGS, but the IHC pattern was consistent with HPV-associated p53 staining and p16 and RNA ISH were positive, and this case was considered to be HPV-A with a secondary *TP53* mutation. In a study on HNSCCs, from 3 to 5% HPV-A tumours harbour *TP53* mutations [36, 37] and Seiwert et al. noted that *TP53* mutations in HPV-A tumours were related to significant tobacco history [37], which was also true for our patient.

The major weakness of this study is the small size of the study cohort. None of the tumours in this study were HPV-I, p53 wt, a molecular group of SCCs that has been described in other sites such as vulva [14, 15, 18] and penis [19]. Double negative (i.e. p16-/HPV- and p53 wt) tumours have been described in the oropharynx (4/110) [21] but are uncommon and more work and larger series where the current recommended IHC interpretation guidelines are applied are warranted to better understand this molecular subtype. The strength of our study is the extensive molecular workup and HPV data on most tumours and that our research group members have previous experience in the accurate interpretation of p53 staining and correlation of IHC with *TP53* sequencing results in SCC. The favourable prognosis of patients with HPV-associated OPSCC has been shown in large studies as reviewed above. Our case series was not designed to assess prognosis, nor is it powered to do that. We can, however, show that p53 IHC is a good surrogate marker for *TP53* mutations and shed light on the potential challenges of p16 interpretation and how performing both p16 and p53 simultaneously can help in the interpretation of both markers, and trigger further molecular testing in approximately 10% of cases.

The current WHO Classification recommends direct HPV detection in geographic areas with low HPV prevalence, when p16 immunostaining is equivocal, when there is a discrepancy between p16 staining and morphology, or when required by clinical trials [2]. Our results show, that performing p53 staining simultaneously with p16 can increase confidence in the interpretation of both immunostains as most HNSCCs show either p16 block positive and p53 wildtype staining in patterns that are specifically associated with HPV, or p16 patchy or negative staining with abnormal (mutant pattern) p53 staining. The basal sparing and null-like p53 staining patterns are only recently described and can be difficult to recognize, but in this small series, when present, were indicators of HPV-A SCC and can aid in interpretation of p16 staining. Other staining results warrant additional testing to ensure accurate classification of tumours as HPV-A or HPV-I. This approach could be especially helpful in centres where mRNA ISH HPV testing is not available and in low-resource settings. p53 IHC is readily available and has a short turn-around time and hence simultaneous testing of p16 and p53 could save time and reduce costs by identifying only a small subset of cases that would need to proceed to HPV testing.

In conclusion, we show that when the current p53 IHC interpretation guidelines are used, abnormal p53 IHC staining patterns shows excellent correlation with *TP53* mutations. Our results indicate that in HNSCC, performing p16 and p53 IHC staining simultaneously can aid in the interpretation of both stains and potentially reduce the need for additional HPV or mutational testing to only selected cases, allowing for accurate subclassification into HPV-associated and HPV-independent tumours, with the potential for further subclassification of the latter into p53abn and p53 wt (Fig. 6). To prove its true clinical potential, a larger study of this dual IHC approach, in patients with OPSCC is needed.

## Data Availability

Data is available at upon reasonable request.

## Code Availability

No custom code was used for this work.
